# A Slippery Slope When Using an Evidence-Based Intervention Out of Context. How Professionals Perceive and Navigate the Fidelity-Adaptation Dilemma—A Qualitative Study

**DOI:** 10.3389/frhs.2022.883072

**Published:** 2022-06-13

**Authors:** Johanna Zetterlund, Ulrica von Thiele Schwarz, Henna Hasson, Margit Neher

**Affiliations:** ^1^Department of Health, Care and Social Welfare, Mälardalen University, Västerås, Sweden; ^2^Department of Learning, Informatics, Management and Ethics, Karolinska Institutet, Stockholm, Sweden; ^3^Center for Epidemiology and Community Medicine, Region Stockholm, Stockholm, Sweden; ^4^School of Health and Welfare, Jönköping University, Jönköping, Sweden

**Keywords:** evidence-based intervention, health care provider, attitude, adaptation, fidelity, parenting education, primary care, Cool Kids

## Abstract

**Introduction:**

Adaptations are often necessary to effectively translate evidence-based interventions (EBI) between contexts, but compliance with the EBIs' core components is still important, which is referred to as the fidelity–adaptation dilemma. In the sustainment phase of implementation, it is the professionals delivering the EBIs who are tasked with the decision-making regarding adaptations, but the currently used models and frameworks mostly focus on the initial phases of implementation. To better understand and guide professionals in using EBIs, there is a need to explore professionals' perceptions of the fidelity–adaptation dilemma. The aim of this study is consequently to explore how professionals perceive and navigate the fidelity–adaptation dilemma when using an EBI out of context.

**Materials and Methods:**

Semi-structured interviews were held with 19 psychologists working in primary care. The interviews concerned EBIs in general and Cool Kids, an evidence-based parenting education program designed for children with anxiety that is now used for children with lower levels of anxiety in another setting. The data were analyzed using an inductive content analysis method.

**Results:**

The analysis resulted in two themes: *My standpoint regarding fidelity and adaptation is clear* and *Managing fidelity and adaptations is complicated*. The first theme summarizes the professionals' perceptions of confidence for either favoring fidelity or adaptations, as well as reasons for why they made adaptations. For the second theme, the professionals expressed concern about sometimes meeting difficulties with the dilemma when following their original inclination and having second thoughts about the impact the adaptations have in practice.

**Conclusion:**

The professionals generally had strong preferences regarding fidelity and adaptations, but neither preference prevented them from facing difficulties with the dilemma. The results point to a need for better information about possible adaptations from developers but also better support and guidance for professionals when implementing EBIs to ensure quality implementation and facilitate implementation. The results of this study can inform the design of support for professionals in managing the dilemma.

## Introduction

It is often complicated to implement interventions that have been proven effective in research, here referred to as evidence-based interventions (EBIs). Models and frameworks are therefore available to facilitate the process and support decision-making regarding adaptations during implementation (1–4). However, the extra resources and guidelines that are present during the initial phases of implementation are often withdrawn when the EBI enters the sustainment phase and is used in practice. Nevertheless, the sustainment phase is still important, as it plays a role in implementation and extensively affects the outcome quality of an EBI (5, 6).

Sustainability is dynamic, and the focus in the sustainment phase should be on the continuous work to find a fit between an intervention and the context since it is always changing (7). According to the dynamic sustainment framework, the concept of sustainability involves ongoing learning and problem solving (7). This dynamic view on sustainability highlights the complexity of implementation and there is, therefore, an ongoing need to work continuously and actively with fidelity and adaptation when implementing an EBI.

An issue that remains to be solved when EBIs are used in the sustainment phase is, therefore, to what extent an EBI needs to adhere to its original plan and whether purposeful adaptations based on restraints and possibilities in the non-research setting, the natural context (8), are acceptable or even desirable. This is commonly referred to as the fidelity–adaptation dilemma (5, 7–10). The definition of adaptation is often defined as any planned, proactive adjustment in the original method to improve the method's fit and effectiveness in the given context (1, 3, 11). Furthermore, context in this matter is referred to as everything that can influence the effectiveness of an EBI that is not part of the intervention (12). Adaptations are often necessary, for example, for an EBI to be effectively translated from one context to another (1, 8, 10), and high fidelity is uncommon (11). Studies report that 44–88% of EBI users make adaptations to the EBI they are working with (1, 3, 13). Common adaptations include the procedure, content, dosage, and target group of the EBI (1, 3, 8, 10). Adaptations have been suggested to be appropriate as long as the core components of an intervention are implemented with high fidelity and the adaptations are aligned with the intervention's goals (8). However, adaptations that are carefully planned and monitored (3, 7) are sometimes not differentiated from adaptations that occur less systematically and without planning (14). These types of adaptations can increase the risk of the intervention becoming ineffective or unsafe (7, 15). Further, even if adaptations are made to improve the intervention's effectiveness, feasibility, and fit, they can still be reactive or affect the EBI's core components (16). Adaptations are therefore sometimes also referred to as any kind of modification that is made, reactive or proactive, planned or unplanned (8). This definition is the one used in this study to allow for the exploration of all types of modifications as they are perceived by professionals, regardless of their timing, or intention.

The responsibility for handling the fidelity–adaptation dilemma and making important and complicated fidelity and adaptation decisions when delivering EBIs in the sustainment phase often lies with the professionals using the EBI in the natural context (5). In many instances, EBIs are recommended by a government body or other external actors without guidelines to the professionals on how to adapt the EBI to fit the new context. This is often due to a lack of knowledge of what to recommend; for example, only one-third of published EBIs in medical care have an adequate description of how to implement them in a natural context (17, 18). This is problematic and can result in a process other than the rational, structured approaches described in most of the available models and frameworks (1–4). Furthermore, the professional delivering the EBI plays one of the most important roles in the implementation (19), and the decisions these professionals have to make related to the dilemma and the dynamic situation they are in can affect not only themselves negatively but also the outcome. Prior research has, for example, illustrated how professionals' characteristics impact how they deal with fidelity and adaptations (5), which indicates that the delivery of EBIs is likely to differ from professional to professional.

Having to deal with the hard decisions and contradictory demands associated with the dilemma can potentially function as a cognitive and ethical stressor among professionals (5, 20, 21). There is a recognized need to further investigate and develop the sustainment phase (22–25). More precisely, there is a need to provide better practical tools to professionals for managing the fidelity–adaptation dilemma (3): first, to facilitate better clinical outcomes, but also to minimize potential stressors among professionals. To do this, more knowledge is needed about the fidelity–adaptation dilemma. There is a research gap regarding how professionals perceive this dilemma, how they manage it, and how they reason about its implications in the sustainment phase (5, 23, 26). Consequently, the aim of this study is to explore how professionals perceive and navigate the fidelity–adaptation dilemma. This was done in the context of an evidence-based parent education program for children with anxiety (Cool Kids), delivered by psychologists but used in a different context and with a different target population than the program was originally designed for.

## Materials and Methods

The study is a qualitative study with semi-structured interviews with psychologists working in primary care in Sweden.

### Case and Study Setting

This study focused on Cool Kids (27), an evidence-based manualized intervention developed in Australia, which was designed to help children being treated for severe anxiety within specialist psychiatric care. It is based on cognitive behavioral therapy and focuses on teaching 7–12 year-old children and their parents how to manage the child's anxiety. The Cool Kids program is group-based and consists of ten 2-h sessions running over a minimum of 10 weeks, and it has been shown to decrease anxiety levels (28). Children and parents receive the intervention in separate groups running in parallel, each led by one group leader with education in psychology.

In 2017, an academic primary care center with a regional commission to disseminate guidance and knowledge about the implementation of evidence-based psychological therapies in primary care recommended their primary care units to use Cool Kids. However, in this case, no specific guidance was given to psychologists working in the primary care units on how to adapt the manual for primary care or children with indications of mental illness, both of which differed from the original treatment as far as the context and target group, respectively. Any situation promoting a decision about fidelity and adaptation was therefore managed by the units and individual professionals.

### Participant Selection

**Figure 1:**
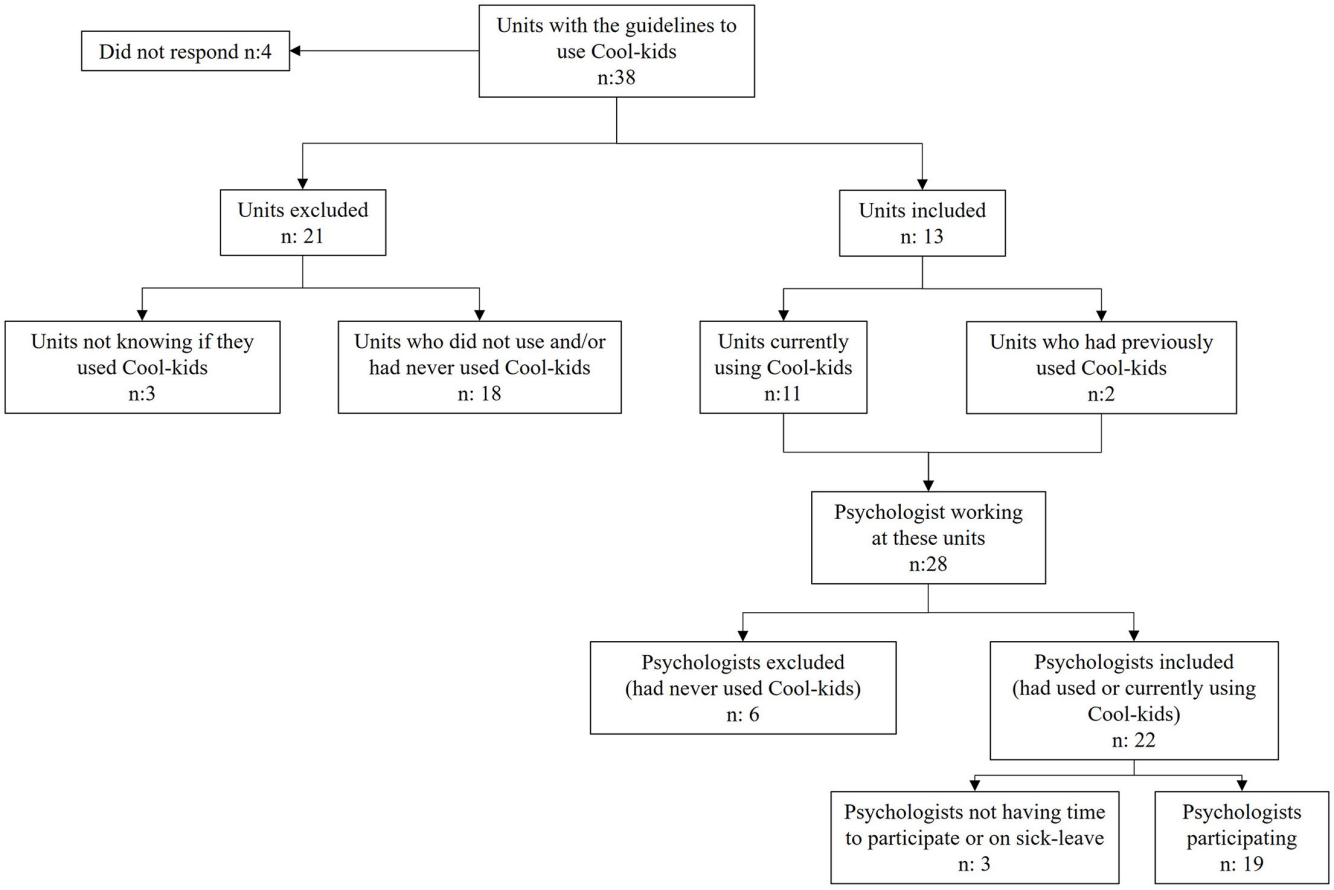
Flowchart of the inclusion process.

Eligible participants were all primary care psychologists experienced with Cool Kids, henceforth referred to as “professionals.” All primary care units that received the recommendation to use Cool Kids by the academic primary care center were contacted (*n* = 38). The inclusion process is illustrated in Figure 1. All psychologists (*n* = 28) in the 13 units that reported that they currently or had previously worked with Cool Kids were invited to participate. Of these, nine psychologists declined to participate because they had never worked with the program, having a busy work schedule, or being on leave of absence. The total sample consisted of 15 women and 4 men between 26 and 65 years old (mean age 39 years). The included professionals had delivered Cool Kids at least once. The professionals varied in their education level; some were newly graduated psychologists (M.Sc. in psychology) and some had doctoral degrees.

### Data Collection

Semi-structured face-to-face interviews were conducted in 2017 by one of two research assistants experienced in interviewing and using EBIs in clinical practice. Fourteen professionals were interviewed individually. Two group interviews (one with two and one with three professionals) were held at the request of the professionals who preferred to be interviewed together with the person(s) they worked with. The interviews lasted 40 min on average (range 26–52 min) and were held in an undisturbed location at the professionals' workplaces. Before the interviews started, the researcher informed the professionals about their involvement in the study, as detailed later in this section.

The interview guide was developed using the dynamic sustainment framework as guidance. This framework acknowledges the continuous work to find a fit between the intervention and the context, which emphasizes that the work with fidelity and adaptation are always present and something to actively address when working with an intervention (7). The questions focused on fidelity, adaptation, and the combination of the two, with the goal of addressing the unexplored aim of this study. The questions explored how the professionals perceived and navigated fidelity, adaptation, and the fidelity–adaptation dilemma, both when using Cool Kids specifically and in general. The interview guide also included questions about whether they had encountered difficulties or obstacles regarding fidelity and adaptations when working with Cool Kids. Examples of questions include: “What are your perceptions of fidelity and adaptations related to Cool Kids?”; “How do you generally address fidelity and adaptations when implementing an evidence-based intervention?” and “Have you experienced difficult trade-offs related to fidelity and adaptations of Cool Kids—if so, tell me more about it.” The interview guide was iteratively refined through pilot testing with both EBI experts and professionals experienced with Cool Kids.

The planning and reporting of this study are in accordance with the Helsinki Declaration, as revised in 2013. The Swedish Ethical Review Authority reviewed the study protocol, concluded that ethical approval was not needed, and provided a statement that they did not have any ethical objections regarding the study (D no. 2017/729-31/5).

All professionals were given oral and written information about the study and a description of what participation entailed, as well as how they could later access the results. The professionals were assured that the researchers would safeguard personal data, that their participation in the study was voluntary and that participation could be withdrawn at any time without reasons given. They were also asked to provide written informed consent before the interviews began.

### Data Analysis

All interviews, the unit of analysis, were audio-recorded, transcribed into text, and analyzed in several steps using inductive content analysis (29). This approach was chosen because the aim of this study was to explore a rather unknown area, and inductive content analysis can provide descriptive insight and increase knowledge about this unexplored phenomenon. With content analysis, it is possible to reduce data to concepts that describe the researched phenomenon—in this case, describing the professionals' perceptions and navigation of the fidelity–adaptation dilemma—by creating categories and sub-categories using collections of codes that share a commonality and themes using collections of categories that are linked through underlying meaning (30).

The themes, categories, and sub-categories were created by condensation and abstraction. This is the process in which the data are shortened from full text into meaning units of words, sentences, or paragraphs containing aspects related to each other through their content or context and codes that label the meaning units. These meaning units and codes are then abstracted into categories, sub-categories, and themes. This process was conducted as follows: To get an overall picture of the content, all the interviews were read and reread by the first author (JZ), while the other authors read a random selection of interviews. The interview data were then uploaded onto NVivo 12 (31), and an initial analysis in which the data were tentatively organized, abstracted, and condensed into meaning units and codes was performed by JZ. The various codes were then compared based on similarities and differences and sorted into categories, sub-categories, and themes. A number of interviews were read and independently coded by another author (MN), and JZ and MN discussed their separate analyses to refine the results. The preliminary results were then discussed by all the authors to achieve a shared understanding of the content, and the final themes, categories, and sub-categories were given titles. Quotes from the interviews were also chosen to illustrate the content of the categories and sub-categories and were translated from Swedish into English. The data are available from the corresponding author upon reasonable request.

## Results

The analysis resulted in two themes: *My standpoint regarding fidelity and adaptation is clear* and *Managing fidelity and adaptation is complicated*. The first theme summarizes the professionals' perceptions of confidence for either favoring fidelity or adaptations, as well as the reasons why they made adaptations. The second theme made it clear that the professionals sometimes, regardless of their preference for either fidelity or adaptations, lacked confidence and encountered difficulties in handling fidelity, adaptations, and the conflict between the two. This theme summarizes how the fidelity–adaptation dilemma affected the professionals. The two themes, together with their categories and sub-categories, are presented and summarized in Table 1.

**Table 1:** Professionals' perceptions of the fidelity–adaptation dilemma—the result of the analysis in themes, categories, and sub-categories.

**Theme**	**Category**	**Sub-Category**
My standpoint regarding fidelity and adaptation is clear	I am certain of my general inclination	The right thing to do is to deliver with fidelity
		Adapting is the right thing for me
	I make adaptations	Delivering with fidelity is impossible
		Delivering with fidelity is not worth it
		Some adaptations do not count
Managing fidelity and adaptation is complicated	It is difficult to manage fidelity and adaptation	It is hard to make adaptations, and it is hard to deliver with fidelity
		Managing trade-offs causes concerns
	What have I done and what has it become?	Adaptations cause uncertainty about the effect
		Adaptations make labeling the EBI confusing

### My Standpoint Regarding Fidelity and Adaptation Is Clear

This theme captures the professionals' preferences of confidence in either favor fidelity or adaptations, as well as the reasons why adaptations occur. These perceptions of feeling certain were summarized in two categories: *I am certain of my general inclination* and *I make adaptations*.

#### I Am Certain of My General Inclination

It was clear that the professionals had strong preferences for what they thought should be done when working with an EBI generally and with Cool Kids specifically. These different standpoints are presented in two sub-categories: *The right thing to do is to deliver with fidelity* and *Adapting is the right thing for me*.

##### The Right Thing to Do Is to Deliver With Fidelity

The professionals talked about the perception that adhering to the original plan or manual of an EBI was the right thing to do. They indicated that adherence should always be the guiding principle. The perception was also that if an intervention was supposed to be evidence-based, or called evidence-based, you should follow the manual step by step as closely as possible.

My attitude is that you should try to follow the manual as much as possible. (Interview 3)

##### Adapting Is the Right Thing for Me

The second sub-category summarizes a perception that directly contrasts the first: following a manual step by step was not the way to work for these professionals. EBIs were perceived as good for research but not necessarily effective in practice. An EBI could serve as a guide, but when the professionals practiced their work, they had to follow what they believed in. They stated that instead of following a manual like Cool Kids strictly, they wanted to do their own thing and do what felt was right and what they were comfortable with as professionals—and that was not to follow a manual to the letter. The perception was that everyone is different, not only patients but also professionals. Each professional may have had a different teaching style, which resulted in different adaptations that fit them and their patients better. As professionals, they also perceived that they knew what helped and what did not and that they had knowledge that a manual did not have. The perceptions were that almost anyone could follow a manual like Cool Kids, but to treat a patient, you also need knowledge beyond what a manual can provide. This perception compelled them to remove parts that they did not believe in or were not consistent with or add things they thought were important or something they wanted to share their expertise in.

I do not believe in one-size-fits-all. I want to be able to work a little more freely with the manual so that it suits me as well. How I want to work, what I want to teach, and like, how I want to progress my work. Some parts of a manual can also be parts that I think are more or less good. And if I do not believe in some parts, some sections, then maybe I do not include it because then I believe that I cannot deliver it in a good way. I have to start with how I want to work as a psychologist and how I see things. (Interview 10)

#### I Make Adaptations

Regardless of whether the professionals believed in or favored delivering with fidelity or making adaptations, they also perceived that there were situations in which adaptations were justified. These perceptions are divided into three sub-categories*: Delivering with fidelity is impossible, Delivering with fidelity is not worth it* and *Some adaptations do not count*.

##### Delivering With Fidelity Is Impossible

The participants indicated that even when professionals wanted to follow the original manual, it was sometimes perceived as an impossible task. Specific working contexts or conditions may require adaptations to be able to deliver the intervention; for example, there could be a limited number of professionals available or a limited amount of space or rooms to gather the participating parents and children, making it impossible to have parents and children in two parallel groups. The professionals also perceived that it was not possible to fully deliver with fidelity because you had a person in front of you for whom you had to adapt the intervention. The perception was that one format does not fit everyone, maybe not even anyone, and you have to make adaptations to make it fit the patient's needs. This could be due, for instance, to a child having two separate diagnoses, making it impossible to treat the patient in a group situation.

So, if I had not made the adjustments, I would not have been able to work with it at all because it is meant to be done with two [group leaders]. (Interview 11)

##### Delivering With Fidelity Is Not Worth It

Another reason for making adaptations was because fidelity took too much effort and “cost” too much compared to what could be gained from following an original intervention plan, which made adaptations the way to go. This could be due to economic costs but also to the fact that the waiting list for care got longer if you followed the original intervention. This made professionals step away from the original manual and instead make adaptations; to deliver with fidelity came at a cost that was too high.

I mean, it demands… then maybe it demands two, at least two people. And then you start counting on how many visits are lost. Then perhaps, in the end, the conclusion is that it is not worth it because we must prioritize the patients we already have. (Interview 14)

##### Some Adaptations Do Not Count

Some adaptations were not perceived as adaptations or were justified or minimized when professionals talked about them. In contrast to the adaptations that were perceived as unavoidable, these adaptations were dismissed as negligible and too insignificant to even be counted as adaptations. From this, it was perceived as possible to retain the perception of delivering with fidelity even though adaptations were made, for example, if adaptations were based on the theory of the program or if parts were only added from other EBIs. Adaptations that were perceived as small or that were not believed to affect the outcome did not count, either. Adaptations were also justified if the professionals knew or believed that others had made the same adaptations. Professionals expressed that they perceived adaptations to sometimes be justified based on the reasoning that the adapted intervention would benefit their patients more than the original intervention as described in the manual.

No, but I still feel like I have followed it quite… quite closely. It is just that I have shortened… Has it been like a minor problem [with the patient], then I have done only three [sessions] [instead of ten sessions]… Actually… I think I have followed the manual, except that I had individual contacts [Instead of group sessions]. (Interview 11)

### Managing Fidelity and Adaptations Is Complicated

In contrast with the clear position the professionals tended to have regarding favoring fidelity or adaptation, more reflective and conflicted perceptions emerged when talking about experiences and consequences of fidelity, adaptation, and the fidelity–adaptation dilemma. These difficulties and questions were perceived by the professionals regardless of their standpoint toward fidelity and adaptation and were divided into two categories: *It is difficult to manage fidelity and adaptation* and *What have I done and what has it become?*

#### It Is Difficult to Manage Fidelity and Adaptation

The professionals expressed a complicated and difficult relationship with both adaptation and fidelity, and they underlined that both delivering with fidelity and making adaptations were riddled with challenges, illustrating the ethical and moral distress that followed. These challenges are summarized in two sub-categories: *It is hard to make adaptations and it is hard to deliver with fidelity* and *Managing trade-offs causes concerns*.

##### It Is Hard to Make Adaptations and It Is Hard to Deliver With Fidelity

The professionals expressed that working with EBIs and making adaptations, whether forced or not, was difficult. They expressed anxiety about drifting too far away from or losing the original intervention with their adaptations, regardless of why the adaptations were made. They also perceived that they had to make difficult trade-offs because they did not want to ruin the intervention by making the “wrong” adaptations. Participants expressed feelings of concern about continually making adaptations without being mindful of the adaptations and forgetting to reflect on what they actually did. They also perceived that they had professional autonomy in their work, which was a good thing, but at the same time, they missed guidelines and support for how to work. They wished they had support on how to relate to fidelity and adaptations and how to stick to the core components of the intervention. The professionals expressed that they worked in a context that did not give them time to reflect on Cool Kids and the adaptations they had to or wanted to make, which could be exhausting and challenging. They also expressed that they were sometimes forced into a structure that was already set and they had to make the adaptations in an EBI that was already routine in that workplace, which felt wrong.

But I do believe that I am doing it wrong… I do not want to ruin the material or do something that it is not intended to do. But I have still seen… I think that the benefit has been… or that it has still been better that I use the material and make the adaptations that I need. But if it is difficult to make adaptations, the trade-offs? Yes, it is because the children are different. (Interview 9)

##### Managing Trade-Offs Causes Concerns

The professionals perceived a conflict between wanting one thing and having to be pragmatic and do another. They expressed psychological stress when trying to deliver with fidelity but were unable to do so. They thought it was stressful to work with an intervention when they wanted the best for their patients but could not deliver the intervention as it was meant to be delivered. They also had the feeling that what they were doing was wrong when making adaptations they did not want to make, but the option was that or doing nothing at all.

Another concern was that everyone made different adaptations, which could result in unequal care. They expressed concern that the care the patients received was dependent on where they lived and who was treating them. They also thought that both fidelity and adaptations had their pros and cons and that the patients they met were helped by different things. Some patients were helped by fidelity, a strict approach to a manual like Cool Kids, and some were helped by more adaptability. They felt that, on the group level, balancing fidelity and adaptation could be problematic and difficult, and they were apprehensive about providing unequal care. There was also a desire for guidance on how to work with the interventions in the future.

We wish that we got some guidelines on how to use the method because we want everyone in the first line [primary care] to do the same thing: the care you get should not depend on where you live. Ideally, we would like a first-line-adapted Cool Kids treatment, but now it does not exist, and now we have done adaptations ourselves because we feel that we have to… (Interview 8)

#### What Have I Done and What Has It Become?

The last category of the *Managing fidelity and adaptation is complicated* theme describes the professionals' perceptions of ambiguity after having made adaptations to Cool Kids and EBIs in general.

##### Adaptations Cause Uncertainty About the Effect

The professionals perceived that it was hard to know how the adaptations they made altered the effectiveness of the EBI. They hoped or had a sense that the outcome was good but noted that they had no way of knowing if any improvement was due to the method or if it was the person delivering the intervention that had made a difference.

Right. And it is difficult to know… It may well be that the outcome would have been the same with ten sessions as with seven, but it is… it is not possible to know… it is difficult to say anything about, of course, but my feeling is that it would not have been better with ten [sessions]. (Interview 16)

##### Adaptations Make Labeling the EBI Confusing

Finally, the professionals were uncertain how the adaptations affected the core of the program and wondered if they could still call it Cool Kids. Could they write in the electronic health record that they worked with Cool Kids, even after adaptations? Was it still an EBI when adaptations were made? They perceived that they could see results from the “new” intervention after the adaptations but was it still evidence-based? Thus, there was ambiguity regarding the implications of adaptations for both the effectiveness and integrity of the intervention.

It feels like… is this evidence now, or is it mostly something that I have like… you may have started working evidence-based, and then it tends to be adapted more and more… you put your own thoughts on the whole thing too, do you understand? And then you get a little bit away from it. And then I do not know how to think about evidence… should it be very square-like and exactly the same? Or can it be a little more fluid? That is something I think about sometimes… when does it go from being evidence-based to becoming a little more [the psychologist's name] special? (Interview 10)

## Discussion

This study aimed to explore how professionals perceived and navigated the fidelity–adaptations dilemma when working with an EBI out of context. The professionals delivering Cool Kids felt certain in their general inclination: They favored either delivering with fidelity or making adaptations, both to EBIs in general and to Cool Kids in particular (*I am certain of my general inclination*). However, there were times and situations when they thought it was acceptable to make adaptations (*I make adaptations*). The professionals also had feelings of uncertainty; they experienced complexity both in making adaptations and when trying to deliver with fidelity (*It is difficult to manage fidelity and adaptation*) and they were uncertain about the effect their adaptations had on the outcome of the intervention (*What have I done and what has it become?)*.

The results indicate that regardless of the professionals' attitudes toward fidelity and adaptation, the dilemma in sustained use of EBIs was unescapable: The professionals still did not, or could not, avoid adaptations. The theory of cognitive dissonance (32) offers a potential explanation for the contradiction between stated beliefs and behavior. For instance, when our cognitions are inconsistent or dissonant with a behavior, we experience a sense of discomfort or tension, which motivates us to try to reduce the dissonance we experience. According to the theory, when experiencing cognitive dissonance, we can, for example, try to justify the behavior by changing the dissonant cognition, add cognitions to make the dissonant cognition fit the behavior, trivialize the behavior or change the behavior to make it fit the dissonant cognition (33). In the current study, this may be reflected in trivialization of adaptations; when, for instance, someone with a strong belief that high fidelity was important nevertheless made adaptations due to contextual constraints. The professionals described adaptations as inescapable, indicating little room to change behavior to better fit a stated preference. To avoid the discomfort of the behavior–cognition gap, the professionals may change their perceptions of fidelity and adaptation or make justifications for the adaptations they make. This indicates that professionals' perceptions about adaptation and fidelity are an insufficient source of information for understanding how professionals navigated the dilemma and more factors are important to understand and investigate.

The findings from this study show that professionals feel certain in their general inclination toward fidelity and adaptation; they either favored delivering an EBI with fidelity or making adaptations. There may be several reasons why professionals differ in whether they favor adaptation or fidelity. For example, previous research has indicated that level of expertise may influence how adaptations and fidelity are navigated and that a higher degree of expertise makes professionals better at judging whether adaptations or delivery with fidelity is necessary (34). Another possible explanation is that the professionals may value research evidence as a knowledge source differently. In the concept of evidence-based medicine the best outcomes come from the integration of the best available treatment, clinical expertise, and patient values (35). Although these knowledge sources are emphasized as equally important the professionals may differ in which knowledge source they value most, as indicated in the findings of this study. This may lead them to have different attitudes toward fidelity and adaptations. For example, ranking research evidence highly may lead to a preference for fidelity but ranking clinical expertise and professional autonomy higher may lead to favoring or having a more relaxed attitude toward adaptation. An inclination toward high professional autonomy may be particularly likely in the Swedish setting. For example, a study with Swedish physicians found that a large majority of physicians made independent clinical decisions according to their own individual assessments without feeling restricted (36).

The professionals, despite their varied inclinations toward fidelity or adaptation in general, also had feelings of uncertainty. They experienced complexity both when making adaptations and when trying to deliver with fidelity. Hypothetically, the professionals could be exposed to cognitive and ethical stressors in these situations and experience contradictory demands (5, 20, 21), for example, when they want to adhere to the original protocol, but this is not possible due to contextual constraints. It can also be the other way around, and the professionals think that adaptations are appropriate, but they nevertheless feel compelled to adhere, which could also be a stressor. The possible negative impact may be accentuated by the large autonomy the professionals have in their clinical practice and that they feel that they are being on their own in managing the fidelity–adaptation dilemma and the findings indicate that they want help and guidance. A lack of support can cause significant tension for professionals who may not only have a deep respect for research but also for patients' individual and cultural variation (9). To further understand whether the fidelity–adaptation dilemma affects the professionals, more studies are needed.

Another uncertainty the professionals expressed was insecurity about the effect their adaptations had on client outcomes, and the professionals expressed that they had few evaluation tools or systems available to understand the outcomes of the EBI. Previous research has highlighted the need to evaluate the impact of adaptations on a target population to avoid unsafe or ineffective programs (37). Thus, professionals may also need help in evaluating the outcomes of adapted EBIs as a way to understand whether their adaptations are positive for the outcomes they want to achieve (37, 38).

The findings clearly show the challenges that professionals faced when navigating the fidelity–adaptation dilemma, and how they perceived a need for help and guidance to deliver quality care. Giving this support is vital, especially since many of the adaptations the professionals reported doing were substantial and made for several reasons. However, solutions to these challenges should not only be sought at the professional level. Clearer terminology concerning evidence and interventions might provide professionals with a more precise language. For example, the concept of evidence in evidence-based medicine uses three knowledge sources, but in EBI only scientific knowledge is emphasized (39). Denoting interventions like Cool Kids research-supported (40) or empirically supported (41) rather than the more ambiguous term evidence-based (42) might be one such clarification. Designers and evaluators of EBIs can also support professionals by providing more useful information about, for example, core intervention components and patient and other contextual factors that may influence effectiveness (43). Such information is currently missing too often (17, 18). More research that illuminates not only if an EBI has an effect but also how, when, and why it has effects, can further help not only the professionals directly but also the guideline developers. For instance, this sort of information could have helped the organizations in the current study to develop better guidance for how Cool Kids could have been adapted to the new context. The results from this study can illuminate what type of information and recommendations are valuable for professionals when dealing with the fidelity–adaptation dilemma.

However, since adaptations are triggered in the interaction between an EBI and a specific context and contextual factors are not constant, it is unlikely that the fidelity–adaptations dilemma can be fully solved through research and more detailed guidelines and terminology. Instead, it is likely that professionals will nevertheless have to navigate the fidelity–adaptation dilemma through the sustained use of implementation, not least because of the varied preferences toward fidelity and adaptation and the multitude of reasons for making adaptations. Together, this indicates that there is a need for support for the professionals, focusing both on guiding navigation and ensuring quality care. The design and evaluation of such support, aiming to help professionals with decision-making regarding fidelity and adaptations when implementing an EBI, is currently underway (44, 45).

### Methodological Limitations

This is a study representing only one professional group (psychologists), and additional studies with other professional groups, other contexts, and other EBIs are needed. However, the heterogeneity of the sample of professionals may strengthen the credibility and transferability of the study, as professionals varied in age and gender, education level, and length of work experience. The authors have expertise in qualitative methods, implementation science, psychology, and public health, which strengthens the study's credibility. Additionally, to prevent inconsistency in coding and to strengthen the study's dependability (29), one author independently coded the data (JZ). Discussions about the coding process were, however, continuously held with another author (MN), who independently coded some of the interviews to make it possible to check for consistency and to enable discussion of the relevance of the established categories and sub-categories in depth. The coding process was iteratively discussed among all the authors. Finally, a checklist was used to achieve more explicit and comprehensive reporting of the study (46).

This study focused on professionals using a specific EBI, Cool Kids, to better understand perceptions of fidelity and adaptation among professionals with experience using an EBI in a natural context. From this, 21 of the 38 potential primary care units initially approached were excluded since they did not use (or did not know if they used) Cool Kids and 6 of the 28 professionals at the included primary care units had never used Cool Kids, which were also excluded; hence, the findings do not represent the perceptions of professionals who, for various reasons, did not use this EBI. This group *may* include professionals who chose not to work with the EBI because it was not possible to use it without adaptations.

## Conclusion

The professionals varied in their attitudes toward fidelity and adaptation and struggled with several types of challenges when using an EBI, irrespective of their fidelity or adaptation preferences. Regardless of their attitudes or preferences, they perceived a need for adaptations, indicating that there was no escaping the fidelity–adaptation dilemma. Furthermore, the professionals experienced uncertainties when working with Cool Kids specifically and EBIs in general and had a desire for support. The result indicates a need for better information and guidance about how to use or potentially make adaptations to a specific EBI in a non-research setting. This support may be provided directly by the EBI developer during the initial implementation phases, but there is likely a remaining need for support and guidance after the initial implementation process. Finally, the results indicate that the professionals included different knowledge sources when trying to implement an EBI and were affected by other factors that probably influenced and impacted themselves, the fidelity–adaptation dilemma and the implementation. This indicates that professionals' perceptions regarding fidelity and adaptation are an insufficient source of information for understanding how professionals navigate the fidelity–adaptation dilemma; instead, other factors need to be considered.

## Data Availability Statement

The data are available from the corresponding author upon reasonable request.

## Ethics Statement

The study involved human participants was reviewed and approved by the Swedish Ethical Review Authority. The participants provided their written informed consent to participate in this study.

## Author Contributions

UT and HH organized the data collection. JZ wrote the first draft of the manuscript and performed the initial data analysis and continuously discussed the results with MN, who also analyzed some of the data. All authors contributed to the conception and design of the study, discussed the final results of the study, and to the revision, and read and approved the submitted version.

## Funding

The data collection was supported by a grant from Forte: Swedish Research Council for Health, Working Life and Welfare (Grant No. 2014-0303). The writing of this manuscript was supported by a grant from the Swedish Research Council (Grant No. 2016-01261).

## Conflict of Interest

The authors declare that the research was conducted in the absence of any commercial or financial relationships that could be construed as a potential conflict of interest.

## Publisher's Note

All claims expressed in this article are solely those of the authors and do not necessarily represent those of their affiliated organizations, or those of the publisher, the editors and the reviewers. Any product that may be evaluated in this article, or claim that may be made by its manufacturer, is not guaranteed or endorsed by the publisher.
